# Transcriptome Analysis Reveals the Mechanisms of Tolerance to High Concentrations of Calcium Chloride Stress in *Parachlorella kessleri*

**DOI:** 10.3390/ijms24010651

**Published:** 2022-12-30

**Authors:** Xudong Liu, Jinli Zhao, Fangru Nan, Qi Liu, Junping Lv, Jia Feng, Shulian Xie

**Affiliations:** School of Life Science, Shanxi University, Taiyuan 030006, China

**Keywords:** calcium stress, *Parachlorella kessleri*, photosynthesis, salt tolerance mechanism

## Abstract

Salt stress is one of the abiotic stress factors that affect the normal growth and development of higher plants and algae. However, few research studies have focused on calcium stress, especially in algae. In this study, the mechanism of tolerance to high calcium stress of a *Parachlorella kessleri* strain was explored by the method of transcriptomics combined with physiological and morphological analysis. Concentrations of CaCl_2_ 100 times (3.6 g/L) and 1000 times (36 g/L) greater than the standard culture were set up as stresses. The results revealed the algae could cope with high calcium stress mainly by strengthening photosynthesis, regulating osmotic pressure, and inducing antioxidant defense. Under the stress of 3.6 g/L CaCl_2_, the algae grew well with normal cell morphology. Although the chlorophyll content was significantly reduced, the photosynthetic efficiency was well maintained by up-regulating the expression of some photosynthesis-related genes. The cells reduced oxidative damage by inducing superoxide dismutase (SOD) activities and selenoprotein synthesis. A large number of free amino acids were produced to regulate the osmotic potential. When in higher CaCl_2_ stress of 36 g/L, the growth and chlorophyll content of algae were significantly inhibited. However, the algae still slowly grew and maintained the same photosynthetic efficiency, which resulted from significant up-regulation of massive photosynthesis genes. Antioxidant enzymes and glycerol were found to resist oxidative damage and osmotic stress, respectively. This study supplied algal research on CaCl_2_ stress and provided supporting data for further explaining the mechanism of plant salt tolerance.

## 1. Introduction

Salt stress is one of the important abiotic stress factors that affect the normal growth and development of higher plants and algae [1]. At present, most of the plant physiological studies on salt stress focus on NaCl, while calcium stress attracts less attention [2].

The impact of high calcium stress on agricultural production is more and more serious. In the secondary salinization of soil, which is caused by over-irrigation, intensive farming, and industrial pollution, Ca^2+^ has accumulated excessively and accounts for over 60% of total cations [3,4,5]. Crop production has been seriously limited by the high calcium stress in greenhouses [6]. In addition, the high content of calcium ions in karst soil also severely affects the plant community distribution and crop yield [7,8,9,10]. Understanding the response mechanism to high calcium stress is of great significance. It will contribute to clarifying the damage mechanism of calcium stress in plants and develop salt-tolerant varieties by means of genetic engineering [2,11,12].

When living in a high calcium environment, higher plant cells usually passively absorb massive Ca^2+^ [8,9]. The Ca^2+^ will then combine with PO_4_^3−^ to form insoluble precipitation in the cytoplasm. This process will not only affect the effective use of phosphorus but also interfere with normal energy metabolism and physiological signal transmission [8,9]. The study by Singh and Goswami showed that high calcium stress destroyed the structure of the photosynthetic membrane, thus affecting the photosynthesis and growth rate [13]. In addition, excessive calcium ions have been proven to cause severe oxidative damage and osmotic stress in plants, which led to reductions in crop yield [14,15,16]. However, up to now, the effect of high calcium stress on algae is unknown.

The small green algae, represented by *Chlamydomonas reinhardtii* and *Dunaliella salina*, have simple structures and similar cellular metabolic pathways to higher plants. Therefore, they have been the model organisms for studying plant photosynthesis and metabolic regulation [17]. Compared with the higher plants, small green algae have the advantages of simpler culture conditions, smaller space requirements, and shorter culture periods. They have offered a lot of meaningful information to salt-tolerant research of plants [18,19,20]. *Parachlorella kessleri* is one of the typical representatives of small green algae. It has diverse habitats and strong adaptability. Some of the strains can even survive in extremely acidic environments [21]. In the previous study, we found and isolated a strain of *Parachlorella kessleri* FACHB-3316 from a high concentration (3.6 g/L) of calcium chloride solution in the laboratory [22]. These characteristics make the strain a good material for studying plant tolerance to calcium stress.

At present, the genetic regulation mechanism of plant salt tolerance is not completely clear, especially at the gene expression level [23]. Transcriptomics can reveal the expression of the whole genome under stress, which is of great significance to increase the understanding of the complex regulatory network related to adaptation and tolerance. Thus, transcriptomics has become an important means of plant salt stress research [24,25,26]. Therefore, the method of transcriptome combined with physiological and morphological analysis was used to explore the high CaCl_2_ alleviation mechanism of *Parachlorella kessleri* FACHB-3316. The purpose of this study is to make up for the deficiency of algal research on calcium stress and provide theoretical data for further explaining the mechanism of plant salt tolerance.

## 2. Results

### 2.1. Physiological Analysis

#### 2.1.1. Algal Growth

**Figure 1:**
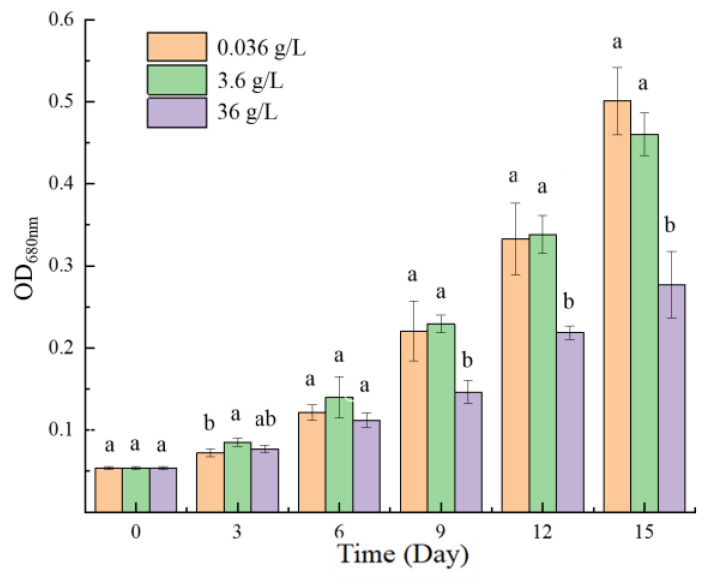
Effects of three concentrations of CaCl_2_ on the growth of *P. kessleri* FACHB-3316. (The OD_680nm_ means the absorbance of the algal solution at 680 nm wavelength. The letters a and b indicate the significance of the difference).

The 0.036 g/L CaCl_2_ group and 3.6 g/L CaCl_2_ group had similar growth rates (Figure 1). In the first twelve days, the cell growth of the 3.6 g/L group was slightly faster than that of the 0.036 g/L group. However, by the 15th day, the 0.036 g/L group accumulated higher biomass. In contrast, the cell density of the 36 g/L CaCl_2_ group was significantly lower than that of the first two groups from the ninth day. In the end, the cell density of the 36 g/L group was about half of the normal group (0.036 g/L CaCl_2_ group).

#### 2.1.2. Photosynthetic Activity

**Figure 2:**
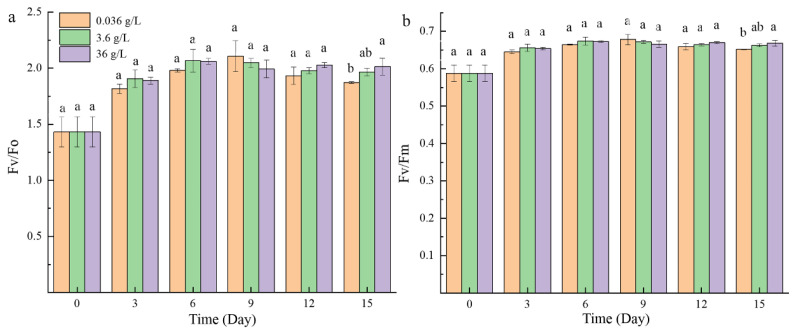
Effects of three concentrations of CaCl_2_ on the photosynthetic activity of *P. kessleri* FACHB-3316: (**a**) Fv/Fo; (**b**) Fv/Fm (The letters a, b and ab indicate the significance of the difference).

The Fv/Fo (Figure 2a) and Fv/Fm (Figure 2b) values of the three groups showed similar changes. After three days of adaptation, the photosynthetic activity of the three groups all maintained in a relatively normal range (about 2.0 for Fv/Fo and 0.65 for Fv/Fm) during the subsequent culture period. On the last day, the Fv/Fo and Fv/Fm values of the 36 g/L group were slightly higher than the normal group.

#### 2.1.3. Chlorophyll Content

**Figure 3:**
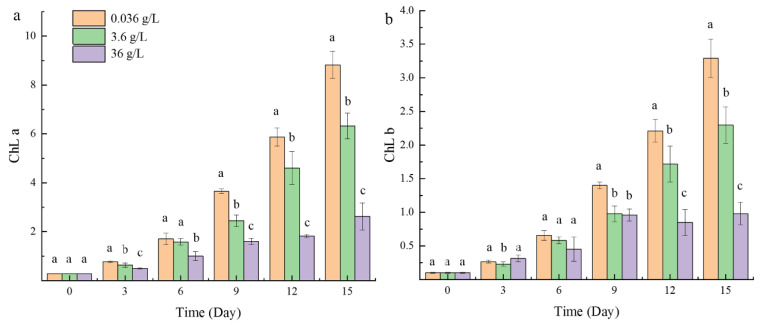
Effects of three concentrations of CaCl_2_ on the Chlorophyll content of *P. kessleri* FACHB-3316: (**a**) chlorophyll a; (**b**) chlorophyll b (The letters a, b and c indicate the significance of the difference).

In contrast to the chlorophyll fluorescence activity, the chlorophyll content of the three groups was significantly different (Figure 3). The contents of chlorophyll a and chlorophyll b in the normal group were significantly higher than those in the 3.6 g/L group, while the 36 g/L group was the lowest. With the increase in time, the difference between the groups became more obvious. On the last day, the chlorophyll a and chlorophyll b content in the normal group was about three times that of the 36 g/L group.

#### 2.1.4. Antioxidant Enzyme Activity

**Figure 4:**
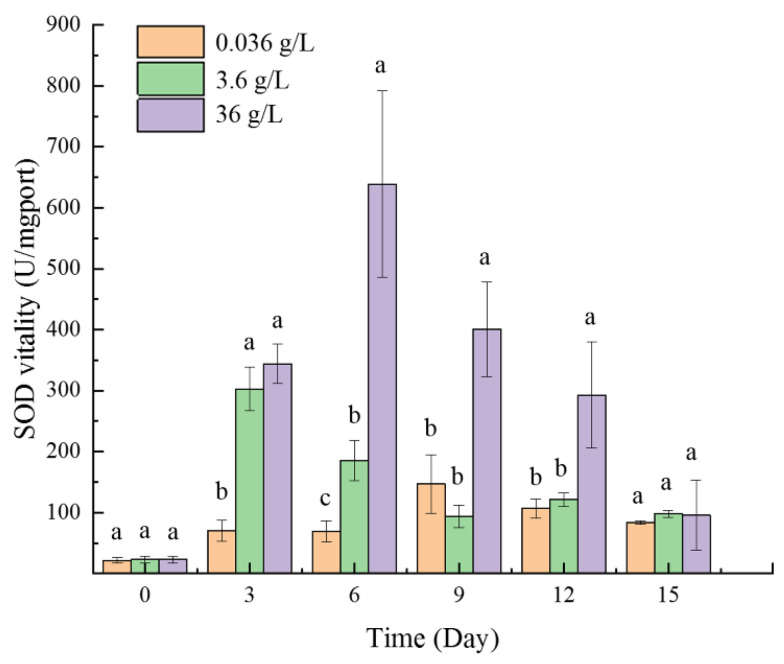
Effects of three concentrations of CaCl_2_ on the superoxide dismutase (SOD) activity of *P. kessleri* FACHB-3316 (The letters a and b indicate the significance of the difference).

The activity of superoxide dismutase (SOD) in the 3.6 g/L and 36 g/L groups increased at first and then decreased gradually (Figure 4). As a control, the normal group (0.036 g/L CaCl_2_ group) maintained a relatively low value during the whole experiment period. The enzyme activity of the 3.6 g/L group reached a peak on the third day and returned to the normal level on the ninth day. During the third to sixth days, the SOD activity of the 3.6 g/L group was significantly higher than the normal groups. The 36 g/L group reached a peak on the sixth day and returned to the normal level on the fifteenth day. During the sixth to twelfth days, the SOD activity of the 36 g/L group was significantly higher than the other two groups. The most obvious difference occurred on the sixth day, when the SOD activity of the 36 g/L group was about nine times that of the normal group. On the last day, there was no significant difference among the three groups.

### 2.2. Morphological Observation

**Figure 5:**
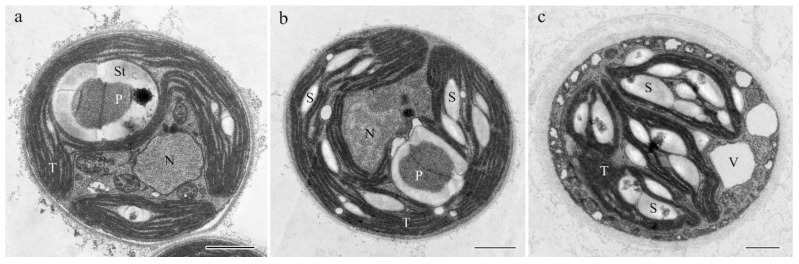
Transmission electron microscopy of *P. kessleri* FACHB-3316: (**a**) 0.036 g/L; (**b**) 3.6 g/L; (**c**) 36 g/L. (T = thylakoids, N = nucleus, P = pyrenoid, S = starch grains, St = starch sheath; V = vacuole, Scale bar 1 μm).

In the 0.036 g/L CaCl_2_ group, the mantel-shaped chloroplasts occupied most of the cell volume and contained an obvious pyrenoid (Figure 5a). Some starch grains were positioned in the thylakoid lamella. Nuclei were lying in a central position. In the 3.6 g/L group, the shape of the chloroplast was regular with more starch grains present (Figure 5b). In the 36 g/L group, a large number of starch granules were positioned in a thinner chloroplast. Many vacuoles of different sizes were present and arranged around the inner side of the cell membrane (Figure 5c).

### 2.3. Transcriptome Analysis

#### 2.3.1. Transcriptome Assembly

**Figure 6:**
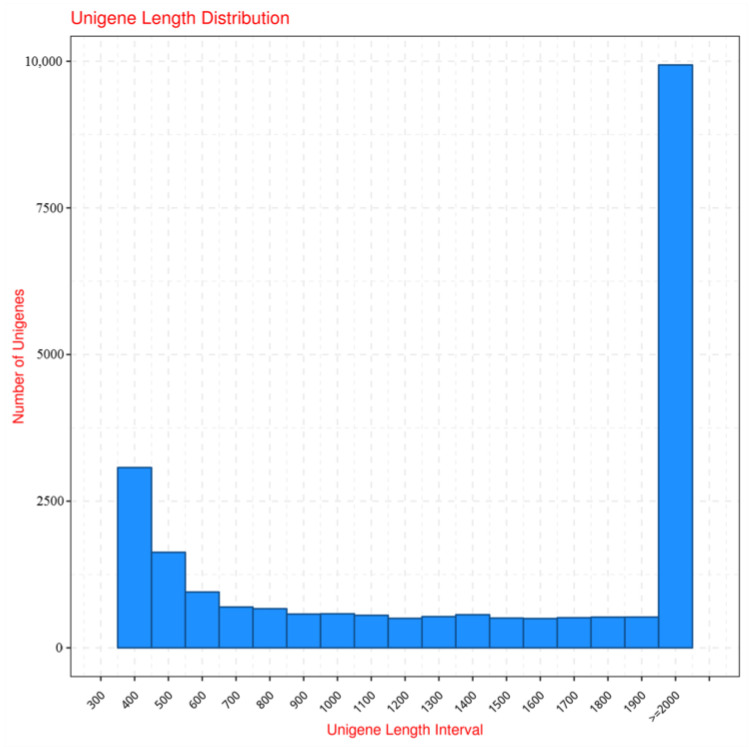
Length distribution of unigenes in *P. kessleri* FACHB-3316.

The data information, including raw reads, clean reads, and clean base numbers, are listed in Table 1. The Q30s quality of all groups was about 93.47~94.44%, and the GC content was similar among groups. After assembling the clean reads, 24,878 unigenes were acquired. The numbers of unigenes with different length intervals are shown in Figure 6, and the splice length distribution is listed in Table 2. The most abundant length interval of unigenes is ≥2000 bp.

#### 2.3.2. Gene Functional Annotation

**Figure 7:**
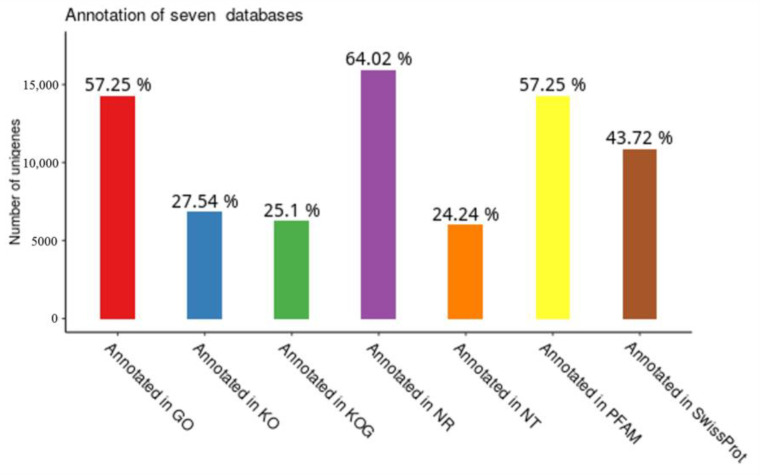
The functional information diagram of unigenes of *P. kessleri* FACHB-3316 in the annotation of seven databases.

**Figure 8:**
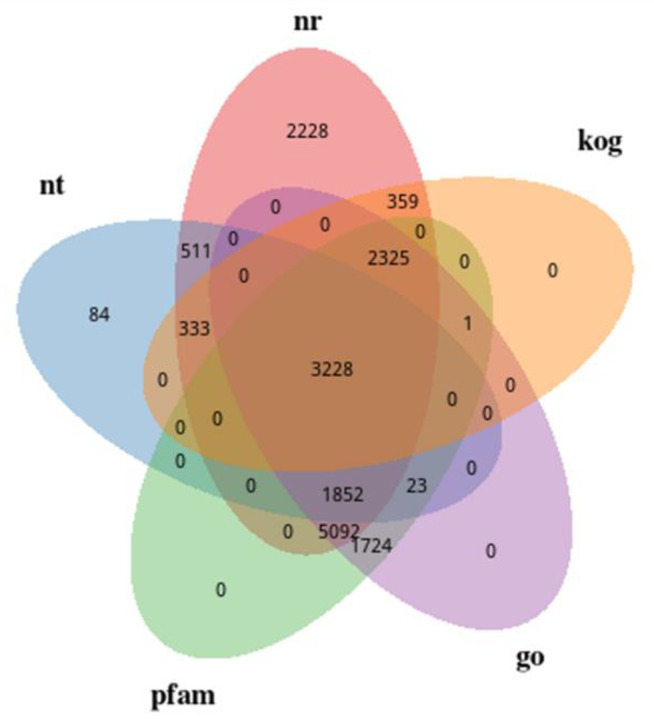
Venn diagram of unigenes annotated in five annotation databases.

**Figure 9:**
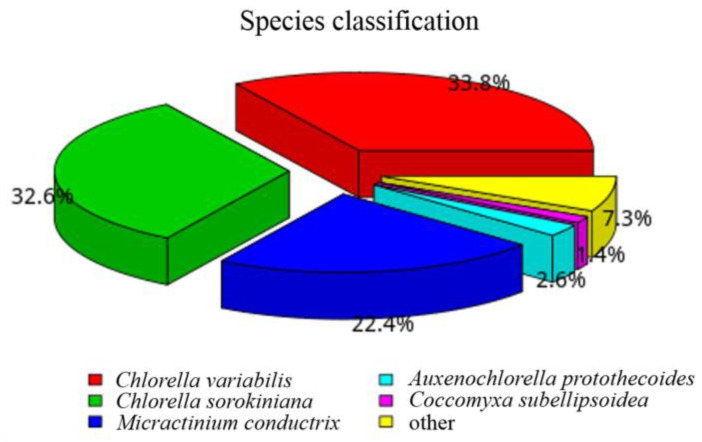
Species diagram of *P. kessleri* FACHB-3316 in NR database annotation.

The annotation of the unigene sequence in the seven annotation databases is shown in Figure 7. A total of 64.02% of unigenes were annotated in NR databases, followed by GO and pFAM with the same proportion of 57.25%. The minimum annotated percentage occurred in NT databases at 24.25%. The proportions of annotated unigenes in Swiss Prot, KO, and KOG databases were 43.72%, 27.54%, and 25.1%, respectively. The Venn diagram showed that 3228 unigenes were shared by the Nr, Nt, Pfam, GO, and KOG databases (Figure 8). Based on the Nr annotation results (Figure 9), *Chlorella variabilis* (33.8%) has the most homologous genes with *P. kessleri* FACHB-3316, followed by *Chlorella sorokiniana* (32.6%), *Micractinium conductrix* (22.4%), *Auxenochlorella protothecoides* (2.6%), and *Coccomyxa subellipsoidea* (1.4%).

**Figure 10:**
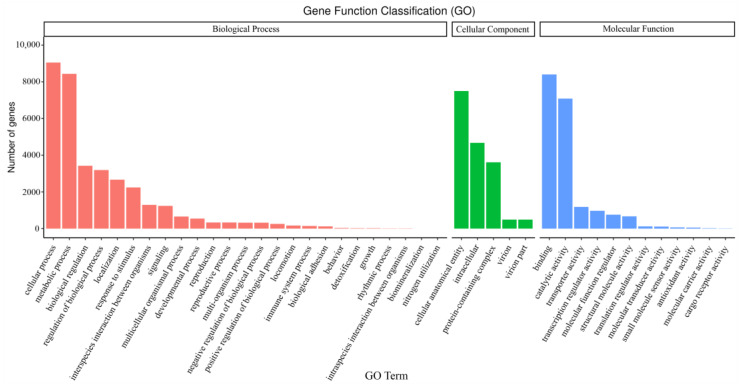
The functional annotation of unigenes in the GO database.

The annotated unigenes in the GO database were sorted into three categories (Figure 10): biological processes (BP, gene number: 34,842), cellular components (CC, gene number: 16,768), and molecular functions (MF, gene number: 19,443). The annotated genes in BP could be further classified into 25 terms. The terms with the most genes were “cellular process” (9059 genes) and “metabolic process” (8437 genes). Similarly, the CC and MF categories were sorted into 5 and 12 terms, respectively. The “cellular anatomical entity” (7497 genes) was enriched by the most genes in CC categories. When in MF, the “binding” (8407 genes) and “catalytic activity” (7084 genes) terms were the most frequent.

**Figure 11:**
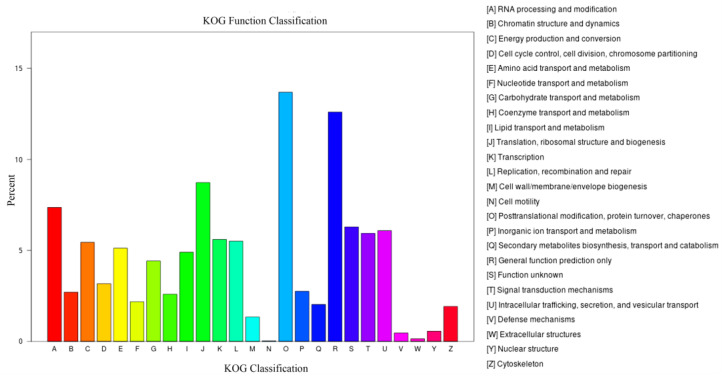
The functional annotation of unigenes in the KOG database.

In the KOG database, the annotated unigenes belonged to 25 functional classifications (Figure 11). Among them, the “Posttranslational modification, protein turnover, chaperones (O)” cluster was the most enriched, accounting for 13.69% of the annotated unigenes. The second most enriched classification was the “General function prediction only (R)” cluster with 12.69% of the annotated unigenes.

**Figure 12:**
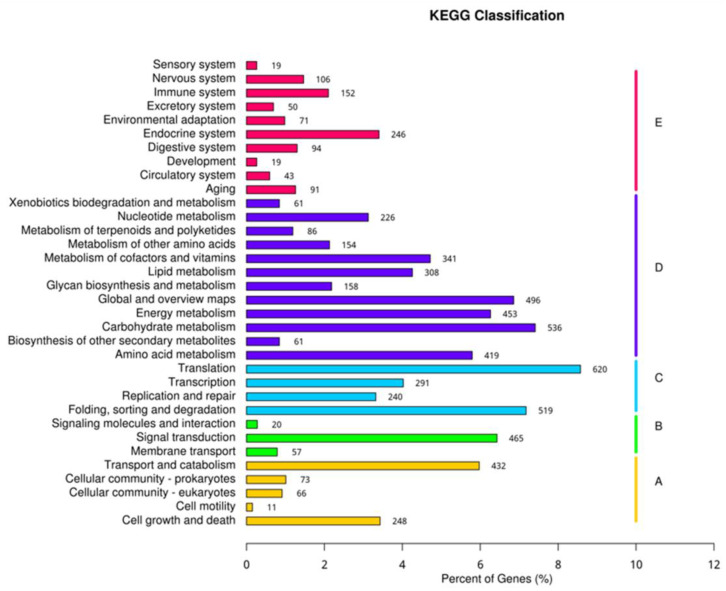
The functional annotation of unigenes in the KEGG database.

The annotated unigenes based on the KO database were further mapped into 34 KEGG metabolic pathways in 5 classifications (Figure 12): cellular processes (A), environmental information processing (B), genetic information processing (C), metabolism (D), and organismal systems (E). The metabolism (D) enriched the most unigenes (3299, 45.61% of the annotated unigenes), and the environmental information processing (A) was the least (542, 7.50%) enriching. The genetic information processing (C), organismal systems (E), and cellular processes (A) accounted for 23.09%, 12.32%, and 11.48% of the annotated unigenes, respectively.

#### 2.3.3. Differential Gene Expression Analysis

**Figure 13:**
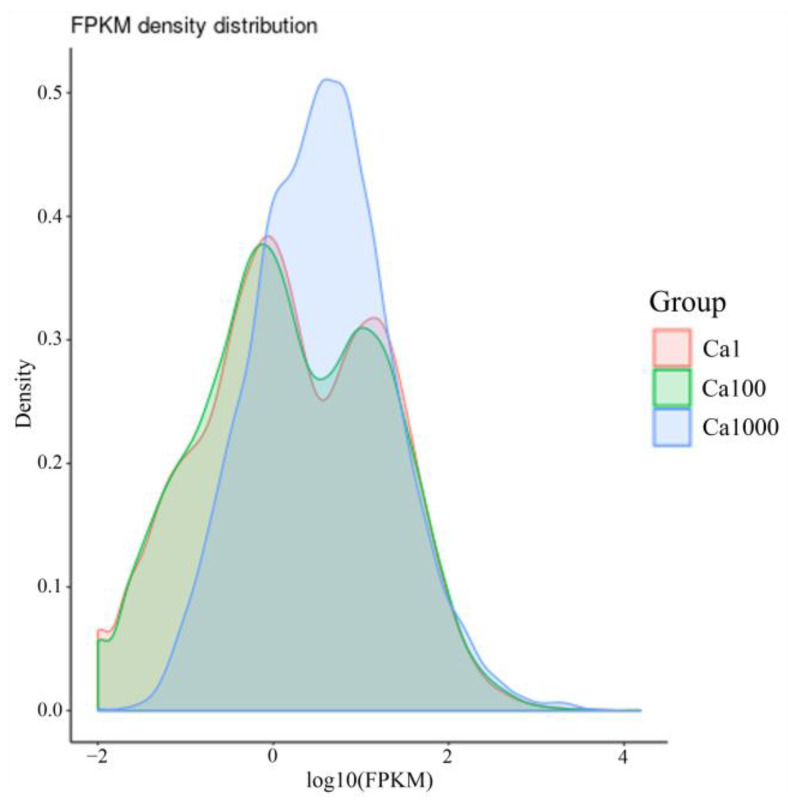
Effects of three concentrations of CaCl_2_ on the gene density distribution.

The FPKM density distribution of three CaCl_2_ concentration groups (Figure 13) showed a similarity between the 0.036 g/L group and the 3.6 g/L group, which were significantly different from the 36 g/L group.

**Figure 14:**
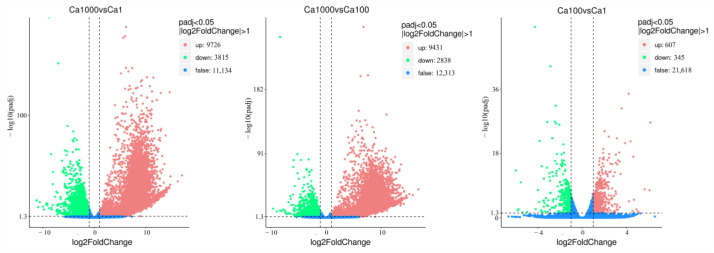
Differential gene volcano map for different concentrations of CaCl_2_ groups.

The volcano map clearly shows the overall distribution of significantly differentially expressed genes (DEGs) between the groups (Figure 14). There are 607 genes up-regulated and 345 down-regulated when the CaCl_2_ concentrate was raised from 0.036 g/L to 3.6 g/L CaCl_2_. When in 36 g/L CaCl_2_, the number of DEGs significantly increased to 9726 up-regulated and 3815 down-regulated genes compared with the normal culture condition, and 9431 up-regulated and 2838 down-regulated genes compared with 3.6 g/L CaCl_2_, respectively.

The top 20 enriched KEGG upregulated metabolic pathways are listed in Table 3 and Table 4. When comparing 3.6 g/L to 0.036 g/L CaCl_2_ (Table 3), a large number of important biosynthesis pathways in eukaryotes were significantly upregulated, including ribosome biogenesis, aminoacyl-tRNA biosynthesis, and many kinds of amino acid biosynthesis (alanine, aspartate, glutamate, valine, leucine, isoleucine, arginine, proline, lysine, histidine, cysteine, methionine, glycine, serine, and threonine). In addition, the photosynthesis-related and selenocompound metabolism pathways were both upregulated. When in 36 g/L CaCl_2_, the algae significantly upregulated the photosynthesis-related pathways.

**Figure 15:**
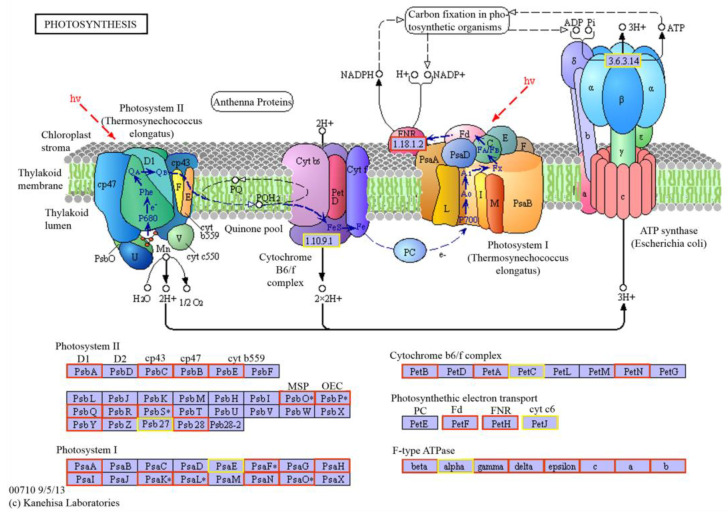
Photosystem and electron transport system pathway in *P. kessleri* FACHB-3316 generated by KEGG. (The red boxes indicate the ko nodes containing the up-regulated differential gene enzymes in 36 g/L CaCl_2_ stress; the orange boxes indicate the ko nodes containing up- and down-regulated differential gene enzymes in 36 g/L CaCl_2_ stress; the * indicate the ko nodes containing the up-regulated differential gene enzymes in 3.6 g/L CaCl_2_ stress).

**Figure 16:**
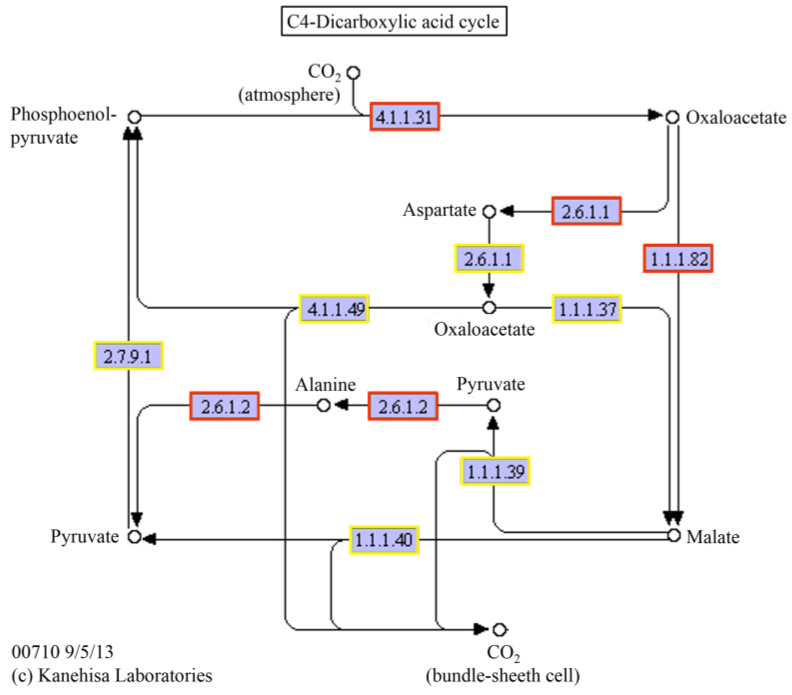
C4-dicarboxylic acid cycle pathway in *P. kessleri* FACHB-3316 generated by KEGG. (The red boxes indicate the ko nodes containing the up-regulated differential gene enzymes; the orange boxes indicate the ko nodes containing up- and down-regulated differential gene enzymes).

**Figure 17:**
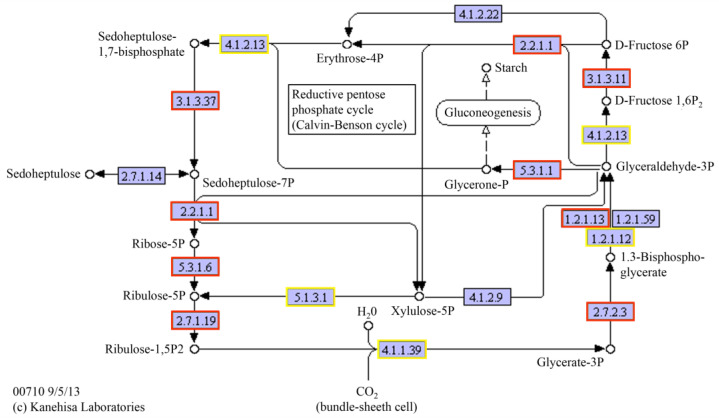
Reductive pentose phosphate cycle pathway in *P. kessleri* FACHB-3316 generated by KEGG. (The red boxes indicate the ko nodes containing the up-regulated differential gene enzymes; the orange boxes indicate the ko nodes containing up- and down-regulated differential gene enzymes).

The photosynthesis metabolism was one of the most enriched KEGG pathways in 36 g/L CaCl_2_ stress. The results showed that 53 genes were significantly up-regulated and 5 genes were down-regulated (*p* < 0.5) in the photosystem and electron transport system process (Figure 15). The up-regulated genes were throughout the whole photosynthesis, including the photosystem II module, photosystem I module, cytochrome b6/f complex module, photosynthetic electron transport, and F-type ATPase module. In the carbon fixation process, a total of 48 genes were significantly up-regulated and 19 genes were down-regulated (*p* < 0.5). The up-regulated genes were distributed in all the pathways of the C4-dicarboxylic acid cycle (Figure 16) and most pathways in the reductive pentose phosphate cycle (Figure 17).

#### 2.3.4. Real-Time Quantitative PCR Analysis

**Figure 18:**
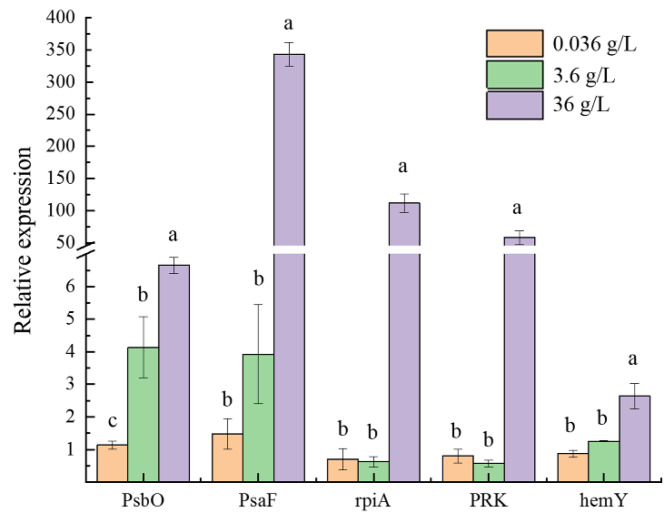
Relative expression levels of related metabolic pathway genes in the 0.036 g/L CaCl_2_ group and the 36 g/L CaCl_2_ group (The letters a and b indicate the significance of the difference).

The expression levels of five key genes (*psbO*, *psaF*, *rpiA*, *PRK*, and *hemY*) were validated by qRT-PCR (Figure 18). The transcript abundance rates were all consistent with the transcriptome sequencing data.

## 3. Discussion

There are many salt-tolerant plants in nature. They have gradually formed a set of well-developed salt tolerance mechanisms in the long evolutionary process. In the previous study, a green alga that could tolerate high concentrations of CaCl_2_ was accidentally found [22]. In this study, the transcriptome combined with physiological and morphological results revealed the mechanisms of tolerance of this strain to high calcium stress, which were mainly by strengthening photosynthesis, activating antioxidant mechanisms, and regulating osmotic pressure.

Photosynthesis offers the matter and energy for the normal growth and development of plants. Salt stress can accelerate the breakdown of chlorophyll a and chlorophyll b, reduce the activity of PSⅡ and PSⅠ, and destroy the components of the thylakoid membrane [27,28]. In this study, with the increase in CaCl_2_ concentration, the chlorophyll content of *Parachlorella kessleri* FACHB-3316 significantly decreased (Figure 3 and Figure 5). This was consistent with the response of cucumber seedlings and tomato seedlings under high Ca(NO_3_)_2_ stress [29]. Li et al. (Li Qingyun) revealed that, compared with high sodium salt, the same concentration of calcium salt could cause a greater decline in the chlorophyll content of strawberries [30]. The possible reason was that high calcium increased the activity of chlorophyllase and loosened the combination of chlorophyll and chloroplast protein, which led to the decomposition and destruction of chlorophyll [31]. However, unlike NaCl stress, the photosynthetic efficiency of PSⅡ did not decrease under the stress of a high concentration of CaCl_2_ (Figure 2). Transcriptome results revealed that the genes involved in photosynthesis were generally up-regulated under a high calcium environment (Figure 15, Figure 16 and Figure 17). When in the 3.6 g/L CaCl_2_, the genes encoding chlorophyll a/b binding protein in the light-harvesting complex (LHC4, LHCB4, and LHCB5) were significantly up-regulated (*p* < 0.5). The overexpression of these genes contributed to the increase in light absorption and the reduction in chlorophyll loss. The genes involving the photosynthesis II oxygen-evolving enhancer protein synthesis (*psbO*, *psbP*), photosystem II 22 kDa protein synthesis (*psbS*), and photosystem I subunit synthesis (*psaF*, *psaK*, *psaL*, *psaO*) were also significantly up-regulated (*p* < 0.5) to maintain the stability of the photosynthetic system. The encoding products of *psbO*, *psbP*, and *psbS* might also participate in the regulation of Ca^2+^ and Cl^−^ [32,33]. When living in the 36 g/L CaCl_2_, the algae massively up-regulated the photosystem genes participating in the synthesis of most electron transport complexes to accelerate energy absorption (Figure 15). The phosphatidylglycerol (PG) synthesis also increased by up-regulating the genes encoding CDP-diacylglyerol synthase (EC: 3.1.3.4) and PGP phosphatase (EC: 3.1.3.27), which contributed to the maintenance of electron transport and thylakoid membrane structure [34]. On the other hand, the genes involved in the C4 cycle and C3 cycle were also significantly up-regulated, which implied an increase in the amount of carbon assimilation. In the C4-dicarboxylic acid cycle, the key gene encoding phosphoenolpyruvate carboxylase (PEP, EC: 4.1.1.31) was up-regulated, which improved the CO_2_ fixation efficiency. The carbon cycle was accelerated by upregulated synthesis of malate dehydrogenase (EC: 1.1.1.82) and alanine transaminase (EC: 2.6.1.2). In the reductive pentose phosphate cycle, key genes encoding phosphoglycerate kinase (EC: 2.7.2.3) and NADP^+^-glyceraldehyde-3-phosphate dehydrogenase (EC: 1.2.1.13) were upregulated, which increased the production of glyceraldehyde-3P (GAP). The ribulose-1, 5-bisphosphate (RuBP) regeneration cycle was accelerated by upregulated synthesis of a series of enzymes, including fructose-1,6-bisphosphatase I (EC: 3.1.3.11), transketolase (EC: 2.2.1.1), fructose-1,6-bisphosphatase II/sedoheptulose-1,7-bisphosphatase (EC: 3.1.3.11, 3.1.3.37), ribose 5-phosphate isomerase A (EC: 5.3.1.6), and phosphoribulokinase (EC: 2.7.1.19). The up-regulation of carbon fixation pathways indicated that the algae increased the carbon fixation efficiency, which was more conducive to the synthesis of carbon-containing compounds. The products would further synthesize the soluble sugar and starch (Figure 5) to complete energy utilization and storage. A similar tolerance mechanism of increasing the efficiency of light and carbon reduction reactions was also found in *Dunaliella salina*, when dealing with high NaCl stress [35]. For *Arabidopsis*, studies revealed that overexpression of photosynthesis-related genes could enhance salt tolerance [36]. Luo et al. found that the stability of the photosynthetic rate was the key to adapting to the high-calcium karst soil for *Cyrtogonellum fraxinellum* [37]. Therefore, we speculated that maintaining stable photosynthesis efficiency and energy input was one of the important mechanisms for *Parachlorella kessleri* FACHB-3316 to resist high calcium stress.

On the other hand, the reactive oxygen species (ROS) balance of the plant will be broken when subjected to salt stress. Excess ROS accumulation can result in severe oxidative damage to plant cells, and further damage important macromolecular substances, such as DNA, protein, and lipids [38,39,40]. Superoxide dismutase (SOD) can first respond to oxidative damage and rapidly catalyze the conversion of the superoxide anion to hydrogen peroxide and dioxygen [41]. Therefore, salt-tolerant plants usually increase the SOD activity to survive in saline conditions [38]. In this experiment, CaCl_2_ stress rapidly induced the increase of SOD activity in *P. kessleri* FACHB-3316. The degree of increase and duration were proportional to CaCl_2_ concentration (Figure 4). This implied that the SOD could rapidly promote the adaptation of algae cells to the high CaCl_2_ environment. The results were similar to the study of calcium stress in cucumber and watermelon [2,42]. In addition, transcriptome results revealed that free amino acid (FAA)-synthesized pathways were up-regulated under 3.6 g/L CaCl_2_ stress, such as proline, alanine, aspartate, glutamate, and so on. Proline was an important adjustment substance for stress resistance in plants. It could react with excessive oxygen radicals to generate harmless substances for plants so as to eliminate the harm of ROS [43] and stimulate the activities of catalase, superoxide dismutase, and polyphenol oxidase [44,45]. The alanine, aspartate, and glutamate metabolism could also help to maintain redox homeostasis [46]. In addition, genes related to the selenocompound metabolism pathway were significantly up-regulated in the 3.6 g/L CaCl_2_ group (*p* < 0.5) for the synthesis of selenoprotein. Trace selenium could not only promote the growth and photosynthesis of algae but also activate the antioxidant defense system to inhibit lipid peroxidation and intracellular ROS formation [47,48]. Therefore, *Parachlorella kessleri* FACHB-3316 could reduce the oxidative damage of CaCl_2_ stress by the synthesis of antioxidant enzymes, free amino acids, and selenides.

Another important mechanism for plants to adapt to salt stress is accumulating osmolytes so as to resist physiological drought, such as soluble sugar, organic acid, and free amino acid [49,50]. In this study, when comparing the 3.6 g/L CaCl_2_ group to the normal group, the DEGs were largely enriched in various amino acid synthesis pathways (Table 3), including proline, arginine, alanine, aspartate, glutamate, valine, leucine, and isoleucine (*p* < 0.5). Zhen et al. also found that Ca(NO_3_)_2_ stress induced the increase in aspartate, glutamine, threonine, serine, glutamate, alanine, and proline contents in leaves and roots of melon seedlings, as well as cystine, histidine, and arginine content in roots [51]. The increasing total free amino acid content was considered a quick response to salt stress in melons [51]. Liu and Wang found that CaCl_2_ promoted the accumulation of free amino acids [52]. In this way, the plant could increase the concentration of the solution to absorb water and nutrients [52]. Among free amino acids, proline was considered to be one of the most effective osmolytes [53,54]. Xiang et al. found that a high concentration of calcium ions could induce a sharp increase in proline content, and therefore maintain osmotic equilibrium in bryophytes [55]. A similar effect of osmoregulation was also found in the alanine, aspartate, and glutamate metabolism [46]. Buayam et al. found that glutamate might provide emergency protection for *Escherichia coli* when the damage to osmotic adjustment ability occurred [56]. Therefore, under the stress of 3.6 g/LCaCl_2_, *Parachlorella kessleri* FACHB-3316 might resist osmotic stress by the massive synthesis of free amino acids. Under the higher stress of 36 g/L CaCl_2_, DEGs were enriched in the glycerol metabolic pathway. Among them, the gene encoding glycerol-3-phosphate dehydrogenase (GPDH, EC: 1.1.1.8, 1.1.5.3), a key regulatory enzyme of glycerol synthesis, was up-regulated. Glycerin was considered the main osmolyte of *Dunaliella salina* [57,58]. By accumulating glycerol, *Dunaliella salina* balanced the osmotic pressure caused by the high salt environment [59,60]. Glycerin was also an important component of cell membranes, which could alleviate the influence on membrane permeability by high calcium stress [61,62]. In *Dunaliella*, glycerol could be synthesized through photosynthesis or degradation of starch metabolic pathways [63,64]. In this study, the large amount of GAP produced by up-regulated photosynthesis could be isomerized into dihydroxyacetone phosphate (DHAP). DHAP further generated glycerol-3P by the catalysis of GPDH, and the latter finally generated glycerol [65]. Therefore, the increases in substrate and enzyme activity might jointly promote the accumulation of glycerol to cope with osmotic stress.

## 4. Materials and Methods

### 4.1. Algal Isolation and Pre-Cultivation

In the present study, the microalga *Parachlorella kessleri* FACHB-3316 was isolated from a 3.6 g/L calcium chloride solution in the lab [22]. It was purified by serial inoculation and expanded in BG11 liquid medium under a light intensity of 60 μmol/(m^2^∙s) and a light: dark cycle of 14 h:10 h [66]. The constant culture temperature was 25 °C.

### 4.2. Experimental Set Up

The precipitated algae cells were inoculated into BG11 liquid medium with three different concentrations of CaCl_2_, that were 0.036 g/L (normal concentration in BG11, marked as Ca1 in transcriptome analysis), 3.6 g/L (100 times the normal concentration, marked as Ca100 in transcriptome analysis) and 36 g/L (1000 times the normal concentration, marked as Ca1000 in transcriptome analysis). Each group included three replications. The culture condition was the same as the pre-cultivation mentioned above.

### 4.3. Physiological Characteristics Determination

#### 4.3.1. Cell Growth

The absorbance of the algal solution at 680 nm wavelength (OD680) was measured every three days by spectrophotometer (TU-1810, Puxi, Beijing, China) to reflect growth status.

#### 4.3.2. Photosynthetic Efficiency

The chlorophyll fluorescence parameters could reflect the photosynthesis status, including light energy absorption, transmission, and consumption. Two parameters, including Fv/Fo (the potential activity of photosystem II) and Fv/Fm (the maximum quantum yield of photosystem II photochemistry), were measured by the portable PAM fluorometer (AquaPen-C AC100, Prague, Czech) every three days.

#### 4.3.3. Chlorophyll Content

The algae were sampled every three days. The absorbance values at A665 and A649 were measured to calculate the contents of chlorophyll a (Chl a) and chlorophyll b (Chl b) as per the method of Mera et al. [67].

#### 4.3.4. Superoxide Dismutase (SOD) Activity

The superoxide dismutase (SOD) activity was also measured every three days following the methods of Ge et al. [68].

### 4.4. Transmission Electron Microscopy Observation

Algae cells of each group were sampled on the fifteenth day and fixed by 3% glutaraldehyde in phosphate buffer overnight at 4 °C. After phosphate buffer washing, 1% aqueous OsO4 in 0.1 M cacodylate buffer was used to fix the samples for two hours, followed by acetone dehydration and Spurr’s resin embedding. The specimens were then made into serial ultrathin sections and stained with uranyl acetate and lead citrate. The ultrastructure was observed by a Hitachi-7700 transmission electron microscope (Hitachi High-Technologies, Tokyo, Japan).

### 4.5. Transcriptome Sequencing and Analysis

#### 4.5.1. RNA Extraction and cDNA Library Preparation

When cultured to the fifteenth day, the algae cells were collected by centrifugation and frozen quickly in liquid nitrogen for RNA extraction.

Total RNA was extracted based on the method of Holmes and Bonner [69]. RNA purity and integrity were evaluated by the NanoDrop 2000c Spectrophotometer (Thermo Fisher Scientific, Waltham, MA, USA) and the Agilent Bioanalyzer 2100 System (Agilent Technologies, Palo Alto, CA, USA), respectively. The construction of mRNA-seq libraries was based on the standard Illumina protocol. The transcriptome sequencing was finished by Beijing Novogene Bioinformatics Technology Co., Ltd. (Beijing, China) using the Illumina HiSeq 2000 Sequencer (Illumina, San Diego, CA, USA), and 150-bp paired-end reads were finally generated.

#### 4.5.2. Data Processing, Functional Annotation, and Metabolic Pathway Analysis

The raw reads were obtained by high-throughput sequencing and processed by filtering the reads containing adapter, poly-N, and low-quality reads to get clean data. The Q20, Q30, GC content, and sequence duplication level of the clean reads were calculated. The transcripts were obtained by splicing clean reads using Trinity and then hierarchically clustered by Corset to get unigenes. The unigene functions were annotated by searching against seven databases, including GO, KO (KEGG Ortholog database), KOG, Nr, Nt, Pfam, and Swiss-prot.

After comparing the clean reads with the reference sequence (Ref, assembled transcripts), the read count information of each sample was acquired by RSEM. Then, the FPKM conversion was performed on the read count to estimate the gene expression level. DEGs among different samples were detected by DESeq [70]. The KEGG pathway enrichment analysis on the DEGs was performed by KOBAS [71].

#### 4.5.3. Quantitative Real-Time Polymerase Chain Reaction (qRT-PCR) Validation

To verify the accuracy of transcriptome results, a qRT-PCR was performed on five genes (*psbO*, *psaF*, *rpiA*, *PRK*, and *hemY*) with smaller *p*-values. The 18S rRNA was selected as the internal control gene. The template was the cDNA library, and the Takara TB Green Premix Ex Taq TM II (Tli RNaseH Plus) was selected as the fluorescent dye. The qPCR reaction system is shown in Table 5, and the primers are shown in Table 6. The amplification procedures were 95 °C for 3 min, 40 cycles of 95 °C for 10 s, 60 °C for 30 s, and 72 °C for 4 s. The expression values were calculated following the method of Pfaffl [72].

## 5. Conclusions

Based on the results, *Parachlorella kessleri* FACHB-3316 revealed the ability of good adaptability to an appropriately high degree of CaCl_2_ stress (3.6 g/L). So, this strain is expected to be applied in high-calcium wastewater treatment. When confronted with higher CaCl_2_ concentration (36 g/L), the growth, pigment content, and morphology of the algae were inhibited and damaged. Maintaining photosynthetic efficiency and improving antioxidant enzyme activity were universal methods for the strain to adapt to all concentrations of high CaCl_2_ stress. Some substances, including selenoproteins, free amino acids, and glycerol, produced effects in different concentrations. The next step in research may focus on the key salt-tolerant genes by combining more means and hopes to provide a molecular theoretical basis for revealing the salt resistance mechanism of plants.

## Figures and Tables

**Table 1:** Summary of Sequencing Data Quality of *P. kessleri* FACHB-3316.

Sample	Raw Read	Clean Reads	Clean Bases	Error (%)	Q20 (%)	Q30 (%)	GC Content (%)
Ca1_1	24,757,741	23,970,283	7.2G	0.03	97.56	93.65	61.1
Ca1_2	23,767,023	22,861,583	6.9G	0.03	97.46	93.47	61.01
Ca1_3	23,361,638	22,330,665	6.7G	0.02	97.91	94.44	61.18
Ca100_1	22,090,363	21,354,250	6.4G	0.02	97.84	94.31	61.25
Ca100_2	23,129,013	22,043,108	6.6G	0.03	97.84	94.31	61.25
Ca100_3	24,195,434	23,402,511	7.0G	0.03	97.71	93.89	61.08
Ca1000_1	23,813,547	23,014,485	6.9G	0.02	97.88	94.48	62.15
Ca1000_2	23,964,132	23,158,172	6.9G	0.02	97.87	94.44	62.18
Ca1000_3	22,363,588	21,575,649	6.5G	0.02	97.92	94.53	62.29

**Table 2:** Splice length distribution of *P. kessleri* FACHB-3316.

	Min Length	Mean Length	Median Length	Max Length	N50	N90	Total Nucleotide
Unigenes (bp)	301	2079	1463	32,200	3446	1018	51,724,505

**Table 3:** The upregulated pathways of differentially expressed genes significantly enriched in KEGG in the 3.6 g/L CaCl_2_ group compared with the 0.036 g/L group.

Pathway Term	Rich Factor	*p*-Value	Gene Number
Ribosome biogenesis in eukaryotes	0.094340	0.000083	15
Aminoacyl-tRNA biosynthesis	0.069182	0.000644	11
Alanine, aspartate and glutamate metabolism	0.056604	0.000865	9
RNA transport	0.094340	0.003755	15
Valine, leucine, and isoleucine biosynthesis	0.031447	0.004667	5
Photosynthesis-antenna proteins	0.031447	0.006148	5
2-Oxocarboxylic acid metabolism	0.044025	0.010170	7
Biosynthesis of amino acids	0.106918	0.015805	17
One carbon pool by folate	0.025157	0.019456	4
Purine metabolism	0.081761	0.021554	13
Photosynthesis	0.044025	0.041502	7
Arginine and proline metabolism	0.037736	0.045134	6
Arginine biosynthesis	0.025157	0.063898	4
Lysine biosynthesis	0.018868	0.078758	3
Selenocompound metabolism	0.018868	0.092687	3
Histidine metabolism	0.018868	0.100006	3
Cysteine and methionine metabolism	0.037736	0.115123	6
Pyrimidine metabolism	0.050314	0.122522	8
Glycine, serine, and threonine metabolism	0.031447	0.128750	5
Steroid biosynthesis	0.018868	0.139764	3

**Table 4:** The upregulated pathways of differentially expressed genes significantly enriched in KEGG in the 36 g/L CaCl_2_ group compared with the 0.036 g/L group.

Pathway Term	Rich Factor	*p*-Value	Gene Number
Photosynthesis	0.024004	0.128858	53
Ubiquinone and other terpenoid-quinone biosynthesis	0.012681	0.163888	28
Ribosome biogenesis in eukaryotes	0.032156	0.188577	71
Porphyrin and chlorophyll metabolism	0.024457	0.195093	54
Alanine, aspartate and glutamate metabolism	0.016757	0.265489	37
Nitrogen metabolism	0.007699	0.282168	17
Folate biosynthesis	0.006793	0.302803	15
Aminoacyl-tRNA biosynthesis	0.022192	0.317240	49
Selenocompound metabolism	0.007699	0.321789	17
Fatty acid elongation	0.004076	0.326930	9
Apoptosis	0.006341	0.336663	14
Cell cycle	0.031703	0.341372	70
Pyrimidine metabolism	0.032609	0.346717	72
Protein digestion and absorption	0.007246	0.354755	16
Biotin metabolism	0.007699	0.362715	17
Base excision repair	0.010417	0.364863	23
Inositol phosphate metabolism	0.010870	0.370652	24
Ether lipid metabolism	0.004529	0.396622	10
Arginine and proline metabolism	0.016757	0.398513	37
Purine metabolism	0.043931	0.402698	97

**Table 5:** The reaction system of qRT-PCR.

Reaction Component	Dose (μL)
1X SYBR Green Supermix	5
Primer-F	0.5
Primer-R	0.5
cDNA	1
ddH_2_O	3

**Table 6:** The primer sequences of qRT-PCR.

Primers	Sequences (5′-3′)
18S-F	TCCAGACATAGTGAGGACAGA
18S-R	ACTCCACCAACTAAGAACGG
PsbO-F	CTGAGCGTTGCACATCAC
PsbO-R	AGACCTCCATGCTGAAGC
PsaF-F	CAAATGCCTTGCTCTCGGA
PsaF-R	GCCCTCACCTTTGGCTTT
rpiA-F	CGGTGTGCTGCTATGAAT
rpiA-R	TCGTCCAATATCCAGCCA
PRK-F	TGCGAGAAGAGAACTCCT
PRK-R	GTGGGAAGGTGTCAAGTG
hemY-F	GATGGTGAAGGTTTGGTA
hemY-R	CATTAGGACCCTCTCAAAG

## Data Availability

Data is contained within the article.
